# Alternative Splicing during Fiber Development in *G. hirsutum*

**DOI:** 10.3390/ijms241411812

**Published:** 2023-07-22

**Authors:** Jing Zheng, Shuhan Wen, Zhipeng Yu, Keyan Luo, Junkang Rong, Mingquan Ding

**Affiliations:** The Key Laboratory for Quality Improvement of Agricultural Products of Zhejiang Province, College of Advanced Agricultural Sciences, Zhejiang A&F University, Linan, Hangzhou 311300, China; jingzheng_0712@163.com (J.Z.); 2021601022025@stu.zafu.edu.cn (S.W.); 13663997428@163.com (Z.Y.); zafulky@stu.zafu.edu.cn (K.L.)

**Keywords:** *Gossypium hirsutum*, alternative splicing, transcriptome, fiber development, GO enrichment, methylation

## Abstract

Cotton is a valuable cash crop in many countries. Cotton fiber is a trichome that develops from a single epidermal cell and serves as an excellent model for understanding cell differentiation and other life processes. Alternative splicing (AS) of genes is a common post-transcriptional regulatory process in plants that is essential for plant growth and development. The process of AS during cotton fiber formation, on the other hand, is mainly unknown. A substantial number of multi-exon genes were discovered to be alternatively spliced during cotton fiber formation in this study, accounting for 23.31% of the total number of genes in *Gossypium hirsutum*. Retention intron (RI) is not necessarily the most common AS type, indicating that AS genes and processes during fiber development are very temporal and tissue-specific. When compared to fiber samples, AS is more prevalent at the fiber initiation stages and in the ovule, indicating that development stages and tissues use different AS strategies. Genes involved in fiber development have gone through stage-specific AS, demonstrating that AS regulates cotton fiber development. Furthermore, AS can be regulated by trans-regulation elements such as splicing factor and cis-regulation elements such as gene length, exon numbers, and GC content, particularly at exon–intron junction sites. Our findings also suggest that increased DNA methylation may aid in the efficiency of AS, and that gene body methylation is key in AS control. Finally, our research will provide useful information about the roles of AS during the cotton fiber development process.

## 1. Introduction

Cotton is an important fiber crop and plays an important role in the textile industry [1]. Cotton fiber is a linear single cell, originated and differentiated from epidermal cells of cotton ovule [2]. The fiber development process is complicated and overlapping, with five stages: cell initiation, rapid elongation, primary cell wall remodeling, secondary cell wall biosynthesis, and maturation [3]. A number of important genes responsible for cotton fiber development were identified with the whole-genome sequencing of the *Gossypium hirsutum* and RNA-seq technology [4,5]. These genes include transcription factors, genes related to the cell wall and phytohormones, and so on [6]. Although various dynamic transcriptome studies have revealed the molecular basis for fiber development, the post-transcriptional modification regulation mechanism of genes related to cotton fiber development is still unknown.

Post-transcriptional modifications include alternative splicing (AS), RNA editing, and mRNA decay [7]. AS is a pre-mRNA process mechanism that enables the production of many transcript isoforms from a single gene, and each transcript can generate a different protein [8]. More than 60%–85% of genes in plants are AS genes [9,10]. Many studies have indicated that AS is highly related to plant defense response, such as high temperature, cold, salt, and drought stress [11,12,13]. AS has also been recently proven to be related to plant growth and development, such as seed germination, circadian clock control, photomorphogenesis, flowing time control, and ovule development in Arabidopsis and rice [14,15,16,17,18,19]. Whether AS event regulation in polyploid crops, such as wheat, cotton, and oilseed rape, shares the same mechanism as the diploid remains unclear compared with the AS analysis in Arabidopsis and rice [20]. A study has shown that AS variation primarily occurs after polyploidization and domestication in wheat [21]. During embryogenesis and grain development in wheat, a global analysis of the regulation of AS in diploid grass and polyploid wheat grains revealed diversity in AS events not only between the endosperm, pericarp, and embryo overdevelopment but also between subgenomes [22]. Researchers have found that AS in cotton occurs in many gene families, such as peroxiredoxin [23] and the MADS-box family of genes [24]. However, a limited number of whole-genome-wide analyses of the AS genes and AS events exist in the cotton species, where in most of them are from diploid cotton [25,26,27] and one is from tetraploid cotton *Gossypium barbadense* [28]. To date, few systematic studies have been conducted on whether AS plays an important role in *G. hirsutum*, especially in cotton fiber development.

AS is mainly regulated by the activity of splicing regulators. Changes in the abundance of these proteins through transcription and AS, post-translational modifications, and interactions with exonic and intronic cis-elements and core elements of the spliceosomes modulate the outcome of pre-mRNA splicing [13]. The AS genes could also be regulated by miRNA, nucleosome occupancy, and DNA methylation [28,29,30]. Little research has been undertaken on the cloning and function of splicing factors in cotton because research on AS in cotton is still in the early stages, and the regulation mechanism of AS is still unclear.

Because little research has been conducted on AS in plant development, particularly in the polar development phase of cotton fiber cell initiation and elongation, the variations and potential roles of AS genes in cotton fiber development are unknown. We used publicly accessible, high-density time point transcriptome data during cotton fiber development to conduct a complete time-course AS analysis in *G. hirsutum*. According to our findings, AS variation occurs often during fiber production in *G. hirsutum*. Furthermore, we discovered large sources of other regulatory mechanisms related to cotton fiber AS in our investigation.

## 2. Results

### 2.1. Statistical Analysis of AS Genes and AS Events

**Figure 1:**
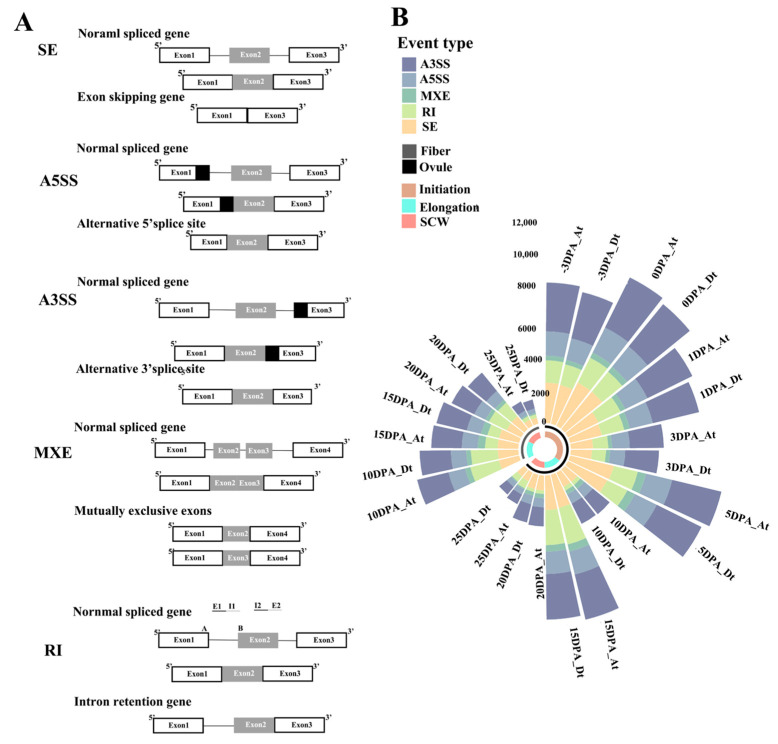
(**A**) Different types of AS events in *G. hirsutum*. (**B**) The bar chart displays the number of the AS events (A3SS, A5SS, MXE, RI, and SE) during cotton fiber development; AS events were statistically identified in ovule samples from nine periods (−3 DPA–25 DPA) and in fiber samples from four periods (10 DPA–25 DPA). The black arc represents the fiber samples, and the gray arc represents the ovule samples. At and Dt subgenomes of *G. hirsutum* were separately counted; Stage 1 Initiation: −3 DPA–5 DPA, Stage 2 fiber elongation period: 10 DPA–15 DPA, Stage 3 secondary wall thickening period (SCW): 20 DPA–25 DPA; skipped exon (SE), Alternative 5′ splicing site (A5SS), Alternative 3′ splicing site (A3SS), mutually exclusive exons (MXE), retained intron (RI)).

The AS events during the ovule and fiber development in *G. hirsutum* were identified by using the transcriptome data with rMATs software (version:4.0.2) [31]. Given that the fiber development is a dynamic and sophisticated development process with overlapping stages, we roughly divided our samples into three stages: namely, the fiber initiation and primary elongation period (stage 1: −3 DPA–5 DPA), fiber elongation period (stage 2: 10 DPA–15 DPA), and secondary wall thickening period (stage 3: 20 DPA–25 DPA). By customizing a user-friendly program, five major AS events were identified, including skipped exon (SE), alternative 5′ splicing site (A5SS), alternative 3′ splicing site(A3SS), mutually exclusive exons (MXE), and retained intron(RI). Figure 1A shows the corresponding types of AS events in *G. hirsutum*. A total of 28,768 pairs of At and Dt homologous genes were extracted via a bidirectional blast and used as the research objects to compare the differences between AS events in *G. hirsutum*.

When the screening thresholds of the AS events were set to 0.1 < PSI (percentage spliced in index) < 0.9 and SJC (skipping junction counts) ≥ 516,283 AS genes (47,751 AS events) were detected during the cotton fiber development in *G. hirsutum*, accounting for 23.31% of the total number of genes in the *G. hirsutum* (Figure 1B and Table 1). Each AS gene produced 2.93 AS events on average. Approximately 41.01% of the total AS genes produced only one AS event. Moreover, the number of AS hotspot genes is 2872, accounting for 17.63% of the total AS genes. These genes could produce more than four AS events during fiber development. Gh_A11G2975 was the most variable splicing gene in *G. hirsutum*, which produced 39 different AS events. Furthermore, 41,253 genes did not have any AS events in all samples, accounting for 71.69% of the total number of *G. hirsutum* genes.

**Figure 2:**
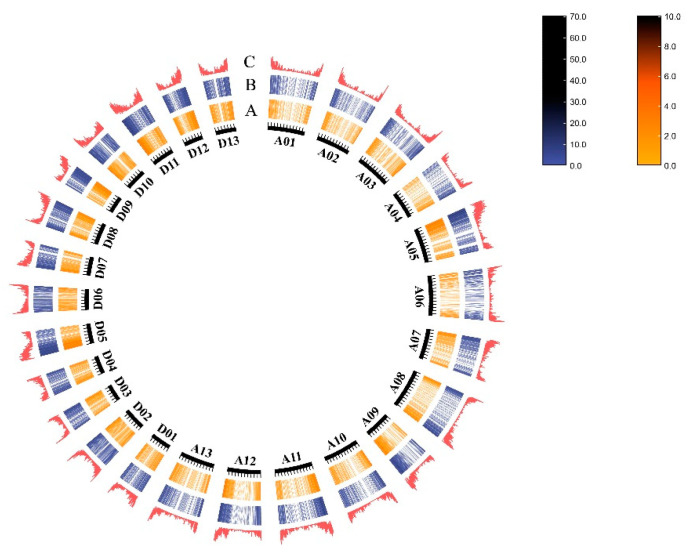
Distribution of the AS genes and AS events across the whole chromosome (**A**: AS events, **B**: AS genes, and **C**: gene densities).

The AS genes and AS events were not evenly distributed across all of the *G. hirsutum* chromosomes. The chrA05 chromosome has the largest number of AS genes and AS events, with a total of 1114 AS genes (3372 AS events). Meanwhile, the chrA04 chromosome has the smallest number of AS genes and AS events, with a total of 308 AS genes (930 AS events). The AS genes and AS events are mainly distributed in the euchromatin region at both ends of each chromosome but less distributed near the centromere in the heterochromatin region (Figure 2).

**Figure 3:**
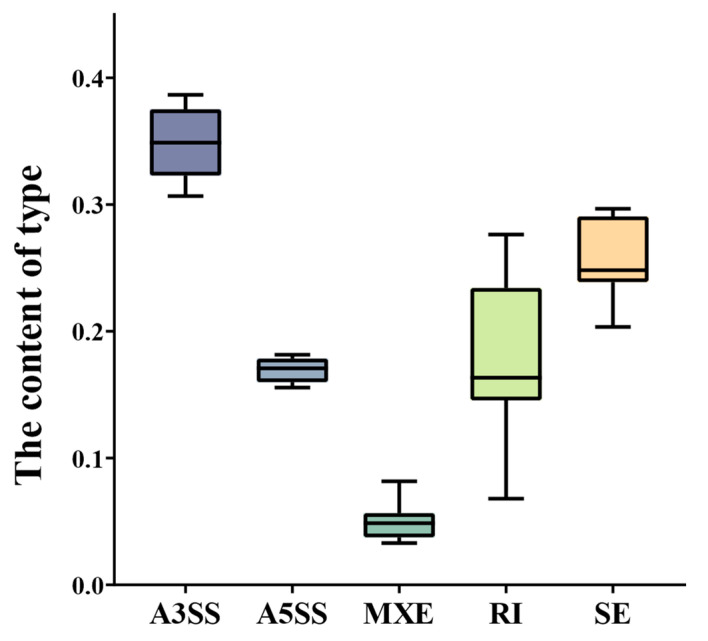
Statistical analysis of different AS events of the samples (skipped exon (SE), Alternative 5′ splicing site (A5SS), Alternative 3′ splicing site(A3SS), mutually exclusive exons (MXE), retained intron(RI)).

AS events can be divided into five types according to the splicing sites: variable 3′-end site (A3SS), variable 5′-end site (A5SS), exon mutually exclusion (MXE), intron retention (RI), and exon jumping (SE). Among the AS genes, 206 AS genes (1.27%) produced five types of AS events (A3SS, A5SS, MXE, RI, and SE), while 8453 AS genes (51.91%) produced only one type of AS event. The number of AS events revealed that the A3SS type was the most abundant AS event type (30.67–38.66%) among all the time course samples of different periods and tissues, followed by SE (20.34–29.67%), RI (6.79–27.64%), A5SS (6.79–27.64%), and MXE (3.29–8.17%). The number of RI types of AS events fluctuated in samples of different periods and tissues, indicating that it has great temporal and tissue specificity (Figure 3).

**Figure 4:**
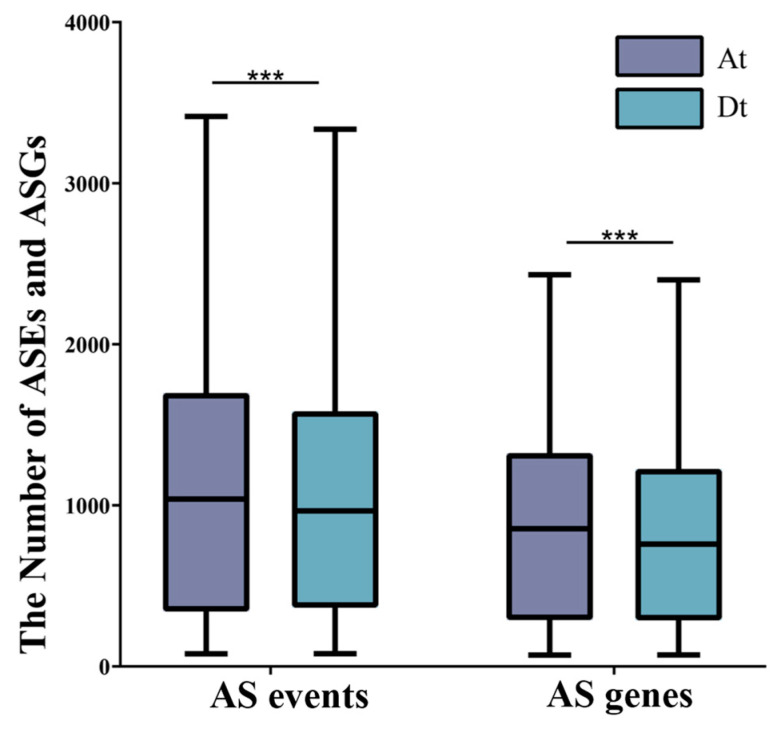
Number of AS genes and AS events between the At and the Dt subgenomes in *G. hirsutum.* This box plot represents the significant difference in the number of AS genes and AS events between At and Dt subgenomes. (*** *p * <  0.001, based on Student’s *t*-test. Skipped exon (SE), Alternative 5′ splicing site (A5SS), Alternative 3′ splicing site(A3SS), mutually exclusive exons (MXE) and retained intron (RI)).

*G. hirsutum* is an allotetraploid that contains At and Dt subgenomes. In this study, the differences between AS genes and AS events were compared between the two subgenomes. Totals of 8, 176 and 8107 AS genes were detected in the At and Dt subgenomes of *G. hirsutum* involving 24,301 and 23,450 AS events, accounting for 28.42% and 28.18% of the total At and Dt subgenomes, respectively. Each At- and Dt-subgenome-splicing gene produced 2.97 and 2.89 splicing events on average, respectively. The statistical analysis showed significant differences in the number of AS genes and AS events among *G. hirsutum* subgenomes. The At subgenome had more AS genes (*p*-value = 8.24 × 10^−11^) and AS events (*p*-value = 2.03 × 10^−13^) compared with the Dt subgenome (Figure 4).

**Figure 5:**
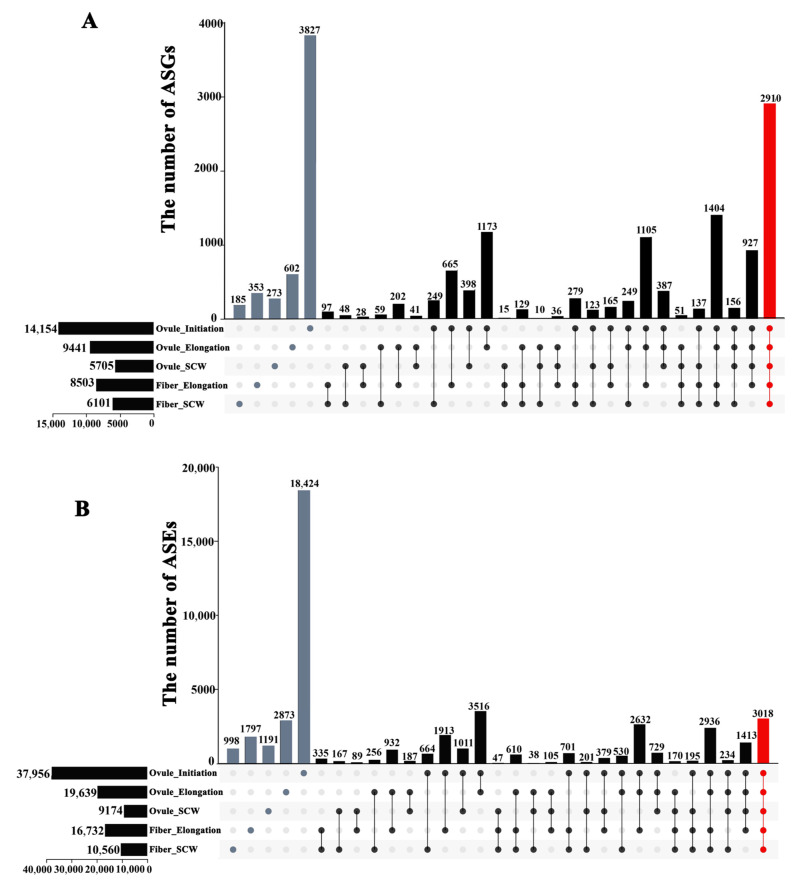
Conservative and specific AS genes (**A**) and AS events (**B**) of different cotton fiber development stages. This Upset plot shows the conservative and specific AS events detected in cotton fiber development stages: ovule initiation, ovule elongation, ovule secondary cell wall biosynthesis, fiber elongation, and fiber secondary cell wall biosynthesis, with the different points representing the AS genes and AS events occurring at each period, red dots representing the number of AS genes and AS events occurring at all five periods, blue dots representing the number of AS genes and AS events occurring at only a single period, and black dots representing the number of AS genes and AS events occurring at 2, 3 or 4 periods.

According to the cotton fiber’s morphological changes, a large number of AS genes and AS events occurred during the three stages of the fiber development of *G. hirsutum*. In ovule samples, 14,154, 9441, and 5705 AS genes (Figure 5A and Supplemental Table S2) involving 37,956, 19,639, and 9174 AS events (Figure 5B and Supplemental Table S3) were identified for stages 1, 2, and 3, respectively. In fiber samples, the number of AS genes and AS events in the ovule samples of the fiber initiation stage (stage 1) was significantly higher than that of the ovule and fiber samples of the fiber elongation (stage 2) and secondary wall thickening stage (stage 3). The 0 DPA ovule samples had the highest number of AS genes (9360) and AS events (19,920), while the 25 DPA fiber samples have the lowest number of AS genes (2363) and AS events (3187).

### 2.2. Identification of the Fiber-Development-Related AS Genes and AS Events

In our study, the AS genes and AS events detected in the ovule and fiber samples at each time point were defined as conservative AS genes and AS events. A total of 2910 conservative AS genes were found, accounting for 13.71% of the total number of AS genes, and 3018 conservative AS events were involved, accounting for 6.32% of the total number of AS events in *G. hirsutum* (Figure 5). The gene ontology (GO) enrichment analysis indicated that these genes are mostly enriched in biological processes (GO terms, such as GO:0006397: mRNA processing, GO:0015031: protein transport, GO:0042752: regulation of circadian rhythm, and GO:0045292: mRNA cis-splicing) via spliceosome (Supplemental Table S4). The large number of universally enriched GO terms indicates that AS is a common phenomenon in fiber development due to the substantial number of genes enriched in various biological process categories.

We discovered that 2384 unique AS genes (3130 AS events) existed in the fiber samples compared with that in the ovule (Supplemental Table S5). The GO enrichment analysis results revealed that these genes can mainly be categorized into GO terms such as “GO:0043412 macromolecule modification, GO:0016192 vesicle-mediated transport, GO:0006468 protein phosphorylation, GO:0016579 protein deubiquitination, GO:0006885 regulation of pH, GO:0006886 intracellular protein transport, and GO:0006511 ubiquitin-dependent protein catabolic process”. The enrichment analysis revealed that the rapid elongation of fibers may be related to the transportation of proteins in the cell, signal transductions, and changes in intracellular pH. Specific AS genes in the above-mentioned biological processes may play important roles in fiber elongation.

We also identified the specific AS genes in different stages of fiber development. A total of 37,956 AS events were detected in the samples at fiber initiation and primary elongation (stage 1), of which 18,424 were specific, involving 9506 AS genes. Moreover, 25,095 AS events were found in the ovule and fiber samples during fiber elongation (stage 2), of which 5602 AS events were specific, involving 4011 AS genes. Approximately 15,664 AS events were found in the samples at the secondary wall thickening period (stage 3), among which 2, 356 AS events were unique to this stage, involving 1868 AS genes (Figure 5).

The specific AS genes in stage 1 were mostly enriched in GO terms, such as “GO:0006396 RNA processing, GO:0006259 DNA metabolic process, GO:0016071 mRNA metabolic process, GO:0044265 cellular macromolecule catabolic process, and GO:0006281 DNA repair”, indicating that genes responsible for the genetic material’s metabolism are prone to be alternatively spliced during stage 1, which may play an important role in fiber initiation (Supplemental Table S6). The specific AS genes in stage 2 were mainly enriched in GO terms, such as “GO:0006644 phospholipid metabolic process, GO:0006654 phosphatidic acid biosynthetic process, GO:0046474 glycerophospholipid biosynthetic process, and GO:0044255 cellular lipid metabolic process”, indicating that AS of the dynamic lipid signal is a key cellular signaling mechanism for fiber elongation (Supplemental Table S7). The GO terms enriched in specific AS genes of stage 3 contained “GO:0043412 macromolecule modification, GO:0006464 cellular protein modification process, GO:0008064 regulation of actin polymerization or depolymerization, GO:0032956 regulation of actin cytoskeleton organization, and GO:0006996 organelle organization”, indicating that protein modification and cytoskeleton organization genes would play important roles in the secondary wall thickening stage of cotton fiber development (Supplemental Table S8).

### 2.3. Regulation of AS in Cotton

The AS is regulated by cis-acting elements within pre-mRNAs and trans-acting factors. The cis-acting elements are the splicing site and the branchpoint sequence that contains partially conserved motifs that are recognized by trans-acting factors. To identify the influencing factors of the AS, we first researched the expression of the pre-mRNA splicing factors (transacting factors) in our samples. Then, the gene length, exon/intron numbers, and GC content of the AS genes and the splicing site were deeply mined.

**Figure 6:**
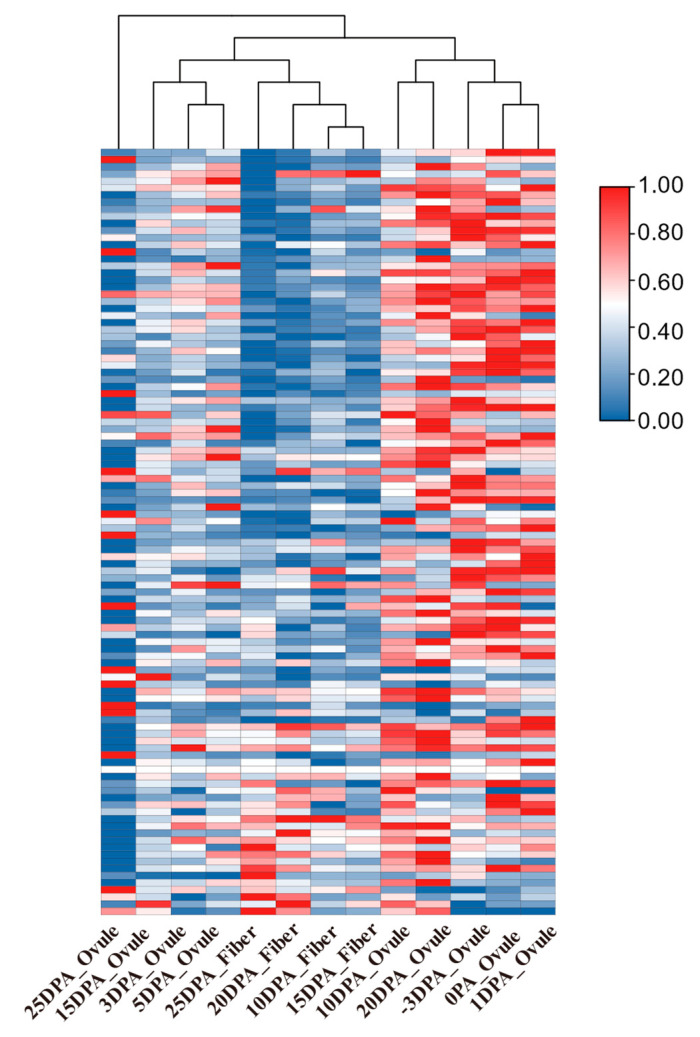
The expression of 108 splicing factors during fiber development in *G. hirsutum*.

We first queried 59 splicing factors in Arabidopsis, which are responsible for mRNA processing, then 108 homologous genes in *G. hirsutum* were identified through the blast. The majority of the gene’s transcription levels remained stable during −3 DPA to 20 DPA. However, many of the splicing factors’ expression was significantly down-regulated in 25 DPA ovules and 10 DPA–25 DPA fibers. This finding is consistent with the result that the number of specific splicing genes in cotton fiber is less than that in the ovule (Figure 6).

**Figure 7:**
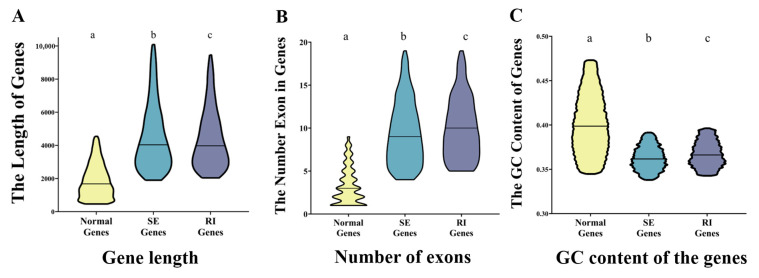
Statistical comparison analysis of the sequence characteristics of AS genes. (**A**): gene length, (**B**) number of exons, and (**C**) GC content of the genes. (a represents Normal genes, b represents Skipped exon (SE) genes and c represent retained intron (RI)genes).

After analyzing the splicing factor, we investigated whether AS genes have a special gene sequence characteristic. To simplify the study, only 3351 “RI group”- and 4896 “SE group”-type AS genes were included for further research. Genes that do not have any AS events were used as the “control group”. These data were statistically analyzed and compared between RI, SE, and normal groups to explore whether AS events have a bias on AS genes’ lengths, exon numbers, GC content of the entire gene, and the splicing site. The results revealed that the average length of normal-group genes was 1868.18 bp, while SE- and RI-group genes were 4517.96 and 4407.29 bp, respectively (Figure 7A). Moreover, each group in the normal group contained an average of 3.30 exons, while every gene in the SE and RI groups contained 9.44 and 10.03 exons on average, respectively (Figure 7B). The average GC content in the normal gene group was 40.15%. The GC contents of the SE and RI groups were 36.27% and 36.74%, respectively (Figure 7C). The statistical analysis showed that the SE and RI splicing events tended to occur in genes with longer sequence lengths, more exon numbers, and lower gene GC content.

**Figure 8:**
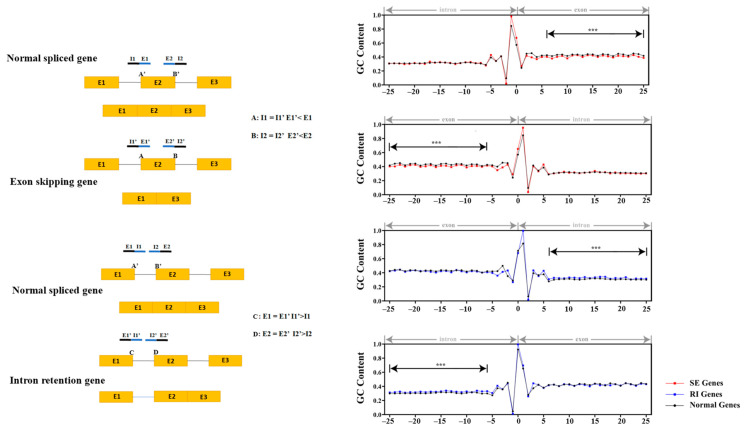
Comparison analysis of the GC content of the junction sites of AS.(*** *p*  <  0.001, based on Student’s *t*-test).

The exon–intron splicing site is important for the correctness of the gene splicing. To explore the relationship between the occurrence of SE, RI, and the sequences near the splicing site, the 25 bp upstream and downstream sequences with a total length of 51 bp were extracted from the splicing site as the center to identify the sequence characteristics of the splicing site. The GC content of the sequence with the length of 20 bp on the intron side of the SE type of genes was not significantly different between the normal group’s introns (I1 = I1′, I2 = I2′) in the two splicing sites involved in the SE type (A’ and B’ splicing points) (A: *p*-value = 0.8553 and B: *p*-value = 0.4432). However, the GC content of the sequence of the SE-type exons was significantly lower than that of the normal type (E1′ < E1, E2′ < E2) (A: *p*-value = 2.51 × 10^−11^ and B: *p*-value = 6.31 × 10^−11^). Of the two splicing sites involved in the RI type (C and D splicing sites), the GC content on the exon side was different from the normal group’s exon (C: *p*-value = 1.24 × 10^−4^ and D: *p*-value = 0.0137). The GC content of the 20 bp sequence on the intron side was significantly higher than the normal group’s intron (I1′ > I1 and I2′ > I2) (C: *p*-value = 1.02 × 10^−8^ and D: *p*-value = 1.44 × 10^−9^) (Figure 8).

Due to significant differences detected in the GC content of the gene body regions and splicing sites between AS genes and normal splicing genes, this study also investigated the DNA methylation levels between the gene regions and splicing sites of AS genes. By analyzing the DNA methylome data of cotton 0 DPA ovule samples, it was found that in the gene body region, AS genes (SE, RI) showed a significant increase in m5C levels compared to non-splicing genes. The level of CpG methylation showed the same trend as m5C, while there was no significant change in the methylation levels of CHG and CHH. At the region of gene splicing sites, the m5C level of the SE type was slightly lower than that of RI type and normal splicing genes. However, the level of CpG methylation exhibited the same trend as the methylation level of the gene body region of AS genes (SE, RI), with higher levels of CpG methylation. Interestingly, the levels of CHG and CHH methylation were significantly reduced compared to normal splicing genes.

### 2.4. Different Types of AS Genes Methylation in Cotton

**Figure 9:**
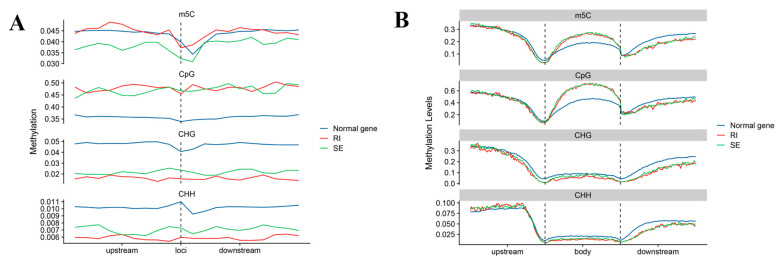
(**A**) Methylation level of the gene body region of AS genes (SE, RI). (**B**) Methylation levels of 50 bp upstream and downstream from the gene splicing sites of AS genes (SE, RI) exon. Skipped exon (SE), retained intron (RI).

Due to significant differences detected in the GC content of the gene body regions and splicing sites between AS genes and normal splicing genes, this study also investigated the DNA methylation levels between the gene regions and splicing sites of AS genes. By analyzing the DNA methylome data of cotton 0 DPA ovule samples, it was found that in the gene body region, AS genes (SE, RI) showed a significant increase in m5C levels compared to non-splicing genes. The level of CpG methylation showed the same trend as m5C, while there was no significant change in the methylation levels of CHG and CHH. (Figure 9A) At the region of gene splicing sites, the m5C level of SE type is slightly lower than that of RI type and normal splicing genes. However, the level of CpG methylation is the same trend as the methylation level of the gene body region of AS genes (SE, RI), with higher levels of CpG methylation. Interestingly, the levels of CHG and CHH methylation were significantly reduced compared to normal splicing genes (Figure 9B).

## 3. Discussion

Because tolerance to abiotic stress is often characterized as polygenic adaptation, many adaptive loci contain genes with subtle effects, and AS has been extensively researched in plant response to abiotic stress. This could be connected to genes that are alternatively spliced at numerous loci and have distinct activities that contribute to abiotic stress [32,33]. In cotton, AS has been investigated in *Gossypium davidsonii*, *Gossypium raimondii*, and *Gossypium barbadense*. Similar in other plants, previous studies have mainly focused on the response of cotton to abiotic and biotic stresses [26,27,28]. The research material of the plant tissue is mainly on the leaf, with fewer studies on the AS genes and events of the ovule and fiber tissues in *G. hirsutum*. In this study, we explored the complicated post-transcriptional regulation during cotton fiber development in *G. hirsutum* via RNA-seq. The considerable AS events suggested that post-transcriptional regulation plays a crucial role in the fiber development process. Our data also indicate that many AS events have taken place in *G. hirsutum* ovules and fibers, which may contribute to the increase of the transcriptome diversity or proteomic complexity, consistent with the previous study [34]. Although AS is largely reported to relate to stress responses in the plant, a number of studies have demonstrated that proper AS of pre-mRNAs and functional genes controlled by the spliceosome is critical for the development of various plant tissues and organs [35,36]. Recently, AS has proven to be involved in JA and ABA signal transductions, indicating that the gene’s AS could participate in the hormone regulation of plant development [9,37,38]. Our study has revealed that the number of AS genes and AS events are highly spatiotemporal and tissue-specific during cotton fiber development. The trend is consistent with the previously defined fiber development stages, such as fiber initiation, elongation, secondary wall thickening stage, and cotton fiber maturation and dehydration stage [3]. The number of AS genes significantly increased at 0 DPA, 5 DPA, and 15 DPA. Given that 0 DPA is the key period in the process of fiber initiation, 5 DPA is the stage of fiber transition from initiation to elongation, and 15 DPA is the timepoint of transition from elongation to secondary wall thickening. Our results suggest that AS may be an important mechanism for *G. hirsutum* to regulate the growth and development of the fiber cells.

In contrast with the results that the number of RI-type AS genes accounts for the vast majority of all the types of AS genes in the relevant studies in cotton, this study found that the A3SS type has the largest number of AS genes, followed by the SE, RI, A5SS, and MXE types. In previous studies, the leaf has been used as the research material. Accordingly, we hypothesize that the distribution of the AS is related to tissues in cotton. In different samples of cotton fiber development, the proportion of RI-type events varies greatly (by up to 4.07 times). In the ovule samples of 10 DPA and 20 DPA, the RI type of AS genes accounted for less than 10%. In the ovules of 15 DPA and fiber samples of 10 DPA and 20 DPA, the RI type of AS genes accounted for more than 20%. The content of RI-type AS events during the fiber development has significant time and tissue specificities. Our results are highly consistent with recent research on AS during fruit development among fresh fruit, and they also have the same finding that the RI type of AS is tissue- and genus-specific [39]. 

AS has proven to be a critical regulatory mechanism for plant development throughout a plant’s entire lifespan [20]. Many genetic and gene function analyses suggest that the AS has been involved in seed dormancy, seed germination regulation, circadian clock control, and phytohormone signaling via *DELAY OF GERMINATION 1 (DOG1)*, *PHYTOCHROME-INTERACTING FACTOR 6 (PIF6)*, *CIRCADIAN-CLOCK-ASSOCIATED 1 (CCA1)*, and *HYPERSENSITIVE TO ABA1 (HAB1)* gene [14,15,40,41]. Although a large number of AS genes and events have been found in cotton by our and the other groups, the key role of AS genes in cotton fiber development is still unknown. Here, the specific AS genes identified in the ovule and fiber tissues are enriched in GO terms highly related to fiber development. Hence, the AS genes’ function and their roles during cotton fiber development must be intensively explored further.

AS is regulated at several levels; to date, multiple sequence-conserved splicing factors and regulators have been functionally characterized in Arabidopsis, which regulate many aspects of development and stress responses [42]. We were able to identify 108 splicing factor genes in *G. hirsutum.* Some of these genes’ lower expression may lead to a smaller number of AS genes being present in cotton fiber samples. Furthermore, the gene structure and GC content may affect the preference for AS. The RI and SE types of AS genes have relatively longer gene lengths, lower GC content, and more exons at the gene level, indicating that AS genes have their characteristics. Another interesting result is that the GC content of the AS genes’ splicing site is different from the constitutive splicing genes, indicating that the junction site’s GC content and DNA methylation level would be important factors.

We discovered that increasing DNA methylation, namely CpG methylation in gene body areas, could improve the efficiency of AS in cotton. Our findings are congruent with those of other crops and plants, including maize and linseed [43,44]. Furthermore, a novel discovery was that the splicing junction’s methylation level contributes to the AS, with high CpG methylation associated with low CHH and CHG methylation, indicating that cis-element regulation via DNA methylation is crucial for gene post-transcriptional regulation such as AS.

In summary, our research has identified an intriguing phenomenon in which many critical functional genes involved in cotton fiber development undergo AS at different phAS events of fiber development. However, whether distinct spliced isoform proteins have diverse activities must be confirmed by cloning each isoform gene and validating overexpression experiments in cotton. Splicing is a complex process involving multiple RNA and protein components. Our findings indicate that during cotton fiber development, the expression of splicing factors significantly regulates the degree of AS. However, it is unclear which splicing factors are the key AS regulatory genes and which types of AS are regulated. In the future, precisely knocking out a splicing factor member with CRISPR-Cas9 could be used to study the biological functions of splicing factors and how to govern cotton fiber growth. Furthermore, our findings suggest that there are links between DNA methylation and AS. The mechanism through which DNA methylation influences fiber development by modulating the AS gene is currently unknown. Using whole genome bisulfite sequencing analysis (WGBS) to study the profile of DNA methylation of AS genes at various stages of cotton fiber development can be a good way to systematically study how DNA methylation maintains correct splicing of cotton fiber genes and thus regulates natural fiber development.

## 4. Materials and Methods

### 4.1. Data Availability

All of the transcriptome data used for this study came from NCBI’s SRA public database (accession numbers in Supplemental Table S1) [5]. In this study, a total of 34 relevant fastq samples were retrieved. The ovule samples ranged from −3 DPA to 25 DPA (−3 DPA, 0 DPA, 1 DPA, 3 DPA, 5 DPA, 10 DPA, 15 DPA, 20 DPA, 25 DPA). Samples of fiber were from the 10 DPA, 15 DPA, 20 DPA, and 25 DPA time periods. The downloads of the reference genome and associated annotation data are available on the CottonGen website [45].

### 4.2. Processing of the Raw Data and Alignment

SRAToolkit [46] was used to download the raw fastq data and filter it using fastp software(version: 0.23.4). The Hisat2 program [47] was then used to align all of the paired-end clean reads to the *G. hirsutum* reference genome. To construct the GTF file to be imported for AS analysis, the resultant SAM files were sorted and assembled using Cufflinks software (version: 2.2.1) [48]. Using CuffMerge tool (in Cufflink), the final GTF data were compared and merged with the original GTF file of the TM1 reference genome.

### 4.3. Performing AS and Statistical Analysis

The AS genes and events were identified with the software, rMATS (version:4.0.2) [31] according to the author’s manual. The IJC (inclusion junction counts) and SJC (skipping junction counts) were used to calculate the PSI values of the AS genes and AS events. AS events with support PSI > 0.1, and PSI < 0.9 and SJC ≥ 5 were retained and counted. Gene ontology enrichment analysis was performed using the OmicShare tools, a free online platform for data analysis (https://www.omicshare.com/).

### 4.4. Gene Expression Analysis

Taking *G. hirsutum* acc. TM-1 (version 2.1) as the reference genome, Htseq-counts [49] were used to calculate the number of reads of a gene in different samples, and the counts of each gene were obtained and normalized to be TPM. The R package, DESeq2, was used for differential expression between samples with the threshold of 2-fold expression changes [|log_2_(fold-change)| ≥ 1] and false discovery rate (FDR) < 0.05. The heatmap of gene’s expression was generated via the tbtools [50].

### 4.5. AS Genes’ Sequence Analysis

In order to analyze the structural and sequence differences of normal splicing genes (genes never undergoing AS) and AS genes in TM-1, the gene length, exon, and intron number, and GC content were extracted and calculated from the GTF file with the in-house Perl scripts. In order to identify the GC content of the splicing junction site, in this study, we defined the splice site as the center, and the upstream and downstream of each 25 bp sequence and the total length of 51 bp were extracted for the in-depth analysis of splice site’s GC characteristics.

### 4.6. Methylation’s Sequence Analysis

The cotton ovules’ (0 DPA) whole-genome bisulfite sequencing (WGBS) data were obtained from the NCBI SRA database (Accession number: GSE61774). The fastp software(version: 0.23.2) was used to filter the raw fastq file [51]. Using Bismark software (version: 0.23.1), the filtered reads were mapped on the *Gossypium hirsutum* TM1 genomic sequences [52]. The methylation level at each site was determined using the calmeth and methGff tool in the Batmeth2 software [53]. A sliding window approach was used to calculate the methylation level of each gene’s gene body region and 2000 bp upstream and downstream regulatory regions (Window size: 0.02 times the gene length or regulation regions, step size: 0.01 times the gene length or regulation regions). The sequences of the 50 bp upstream and downstream splicing sites of SE and RI genes, as well as each exon splicing site of normal splicing genes, were retrieved. These sites’ methylation levels were determined using sliding windows (Window size: 10 bp, Step size: 5 bp).

## 5. Conclusions

The AS genes and events during various phAS events of the development of upland cotton fibers were thoroughly investigated in this study. Although AS occurs regularly throughout the growth of fibers, the genes and events involved in this process were highly spatiotemporally and tissue-specific. With minimal impact on chromosomal length, the distribution of AS genes and AS events on each particular chromosome was strongly correlated with the gene density. A significant number of At and Dt subgenome AS genes and AS events was identified. In addition, we found that AS may be controlled by the way of the splicing factors’ expression and changes in the length, exon count, and GC content of the gene, particularly at the splicing junction location. This research contributes to a better understanding of the gene expression and AS regulatory mechanisms of genes involved in fiber development, as well as the AS profiles of *G. hirsutum*. The discovery of a potential link between AS and the fiber development in *G. hirsutum* may open up a new direction of research into the identification of the molecular mechanisms underlying cotton fiber development in the future.

## Figures and Tables

**Table 1:** Statistics on the number of AS genes in different periods (unit: pieces; skipped exon (SE), Alternative 5′ splicing site (A5SS), Alternative 3′ splicing site (A3SS), mutually exclusive exons (MXE) and retained intron (RI)).

	A3SS	A5SS	MXE	RI	SE	SUM
−3 DPA_Ovule	4369	2343	438	1960	3072	8437
0 DPA_Ovule	4832	2656	563	2339	3642	9360
1 DPA_Ovule	4362	2386	527	2133	2614	8309
3 DPA_Ovule	3253	1664	394	1431	1733	6354
5 DPA_Ovule	4599	2514	799	2176	3374	9237
10 DPA_Ovule	2268	1114	475	453	1316	4605
15 DPA_Ovule	4177	2206	489	3188	2645	8669
20 DPA_Ovule	1977	949	417	371	1240	4091
25 DPA_Ovule	1102	602	141	804	805	2894
10 DPA_Fiber	3040	1636	494	2612	1676	6849
15 DPA_Fiber	3053	1528	434	1215	1675	6067
20 DPA_Fiber	2402	1213	392	1833	1321	5420
25 DPA_Fiber	1053	508	164	411	599	2363

## Data Availability

All sequence data used in the analysis are available from NCBI under PRJNA490626 and PRJNA262137, and all scripts used to analyze the data are available from Multivariate Analysis of Transcript Splicing (MATS) under (Multivariate Analysis of Transcript Splicing (MATS) (sourceforge.net)).

## Supplementary Materials

The following supporting information can be downloaded at: https://www.mdpi.com/article/10.3390/ijms241411812/s1.
